# More Dengue, More Questions

**DOI:** 10.3201/eid1105.050346

**Published:** 2005-05

**Authors:** Scott B. Halstead

**Affiliations:** * Pediatric Dengue Vaccine Initiative, Rockville, Maryland, USA

**Keywords:** Dengue, Aedes albopictus, viral hemorrhagic fever

Dengue is epidemic or endemic in virtually every country in the tropics; it is even cited in the Guinness World Records, 2002, as the world's most important viral hemorrhagic fever and the most geographically widespread of the arthropodborne viruses. As illustrated in this issue of Emerging Infectious Diseases, dengue epidemics are expanding rapidly, as is the literature on the subject. That dengue was transmitted in the United States for nearly 1 year in 2001 (*1*) should serve as a wake up call to Americans, most of whom are ignorant of the threat of this disease. Both the major dengue vectors, *Aedes aegypti* and *Ae. albopictus*, are widely distributed in the continental United States.

This emerging disease continues to baffle and challenge epidemiologists and clinicians. Despite endemicity of 3 or more different dengue viruses, why does severe dengue occur in some populations and not in others? Why are children principally affected in some areas and adults in others? How can severe dengue reliably be recognized early enough to permit appropriate therapy to be applied? Recent studies point in the direction of answers to these questions.

During an infection with any of the 4 dengue viruses, the principal threat to human health resides in the ability of the infecting virus to produce an acute febrile syndrome characterized by clinically significant vascular permeability, dengue hemorrhagic fever (DHF). However, because at onset vascular permeability exhibits only subtle changes, how can a diagnosis be made early enough to begin life-saving intravenous treatment? In persons with light skin color, the standard syphygmomanometer cuff tourniquet test has been widely used to screen children in outpatient settings; a positive test result is an early warning of incipient DHF. Because of genetic diversity among humans, the tourniquet test as a screening tool requires widespread evaluation and validation. In a prospective study of 1,136 Vietnamese children with serologically confirmed overt dengue infections, the tourniquet test had a sensitivity of 41.6%, a specificity of 94.4%, a positive predictive value of 98.3%, and a negative predictive value of 17.3% (*2*). A positive result should prompt close observation, but a negative result does not exclude an ongoing dengue infection.

A more robust screening test could result from the studies of Wills et al., who measured the size and charge characteristics of proteins leaking through the endothelial sieve in DHF patients (*3*). Such changes are caused, presumably, by a cytokine cascade, as yet incompletely identified. Their observations suggest that capillaries leak in most overt dengue infections, but fluid loss is not at levels that alter cardiovascular status. Quantitative differences in duration and amount of protein leak demarcate DHF from dengue fever. Importantly, the authors found increased amounts of serum proteins, increased differential protein excretion, and increased amounts of heparan sulfate in the urine of DHF patients. Detecting protein or heparan sulfate in acute-phase urine could provide early evidence of increased vascular permeability.

Humans are not uniformly susceptible to the DHF syndrome. HLA gene distribution correlates with increased susceptibility as well as with increased resistance (*4*). In addition, a powerful resistance gene is found in blacks (*5*). Importantly, susceptibility to vascular permeability during a dengue infection is age-related. This conclusion comes from a study of the age distribution of patients hospitalized during the 1981 DHF epidemic in Cuba. In that epidemic, persons 2–50 years of age were exposed to infections with dengue 1 virus (DENV-1) from 1977 to 1979 and DENV-2 virus in 1981 at similar rates (*6*). Thus, age-specific hospitalization and death rates were a measure of intrinsic susceptibility to vascular permeability during secondary DENV-2 virus infections. The youngest children were found to be at greatest risk; rates fell rapidly and were lowest in older teenagers and young adults, rising again somewhat in older patients. The susceptibility of young children to DHF precisely paralleled age-related changes in microvascular permeability measured in normal children and adults.

That cytokines, not virus, damage endothelial cells during the course of a dengue infection could be inferred from a recently published immunopathology study of human tissues from DHF patients (*7*). Despite in vitro evidence that dengue viruses infect human endothelial cells, dengue virus was found to have replicated in vivo in monocytes, macrophages, and B lymphocytes. While endothelial cells stained for dengue antigens, this staining was concluded to be the result of in vivo deposition of virus-antibody complexes but not cellular infection.

Differences in intrinsic virulence are widely believed to explain the ability of some dengue virus strains to cause DHF while others do not. Indeed, phenotypic attributes of avirulent American DENV-2 genotype viruses appear to correlate with differences in disease severity (*8*). A different explanation was provided by a prospective fever study in Iquitos, Peru. In 1990, DENV-1 virus entered Iquitos and became endemic. In 1995, widespread, mostly silent infections with American genotype DENV-2 were detected. Many persons infected with DENV-2 had antibodies elicited by an earlier DENV-1 infection. In contrast to Cuba, DHF did not develop in any of these secondarily infected persons. Antibodies to DENV-1 in the sera of Iquitos residents strongly neutralized American genotype DENV-2 viruses, but not virulent Asian genotype DENV-2 viruses isolated from DHF epidemics in the Americas (*9*). DENV-2 antigenic differences, reflected in heterotypic neutralization by antibodies to DENV-1, appeared to result in downregulated infection severity and corresponding clinical expression.

In the American tropics, with the exception of Cuba in 1981, most dengue cases were observed initially in adults. An illustration of this phenomenon is exhibited by the recent outbreak in Hawaii, where primary infections with DENV-1 virus produced dengue fever syndrome in adults. Dengue fever syndrome in susceptible adults may be contrasted to the innate susceptibility of children for vascular permeability syndrome during a secondary dengue virus infection. In Southeast Asia, the epicenter of DHF epidemics in children, dengue infection rates are falling, resulting in changing epidemiologic patterns of DHF. In Thailand, for example, the modal age at which children are hospitalized for DHF has steadily increased over the past several decades (A. Nisalak, pers. comm.). In addition, because an increasing number of persons experience their first dengue infection at an older age, dengue fever cases are now appearing in adults.

**Figure:**
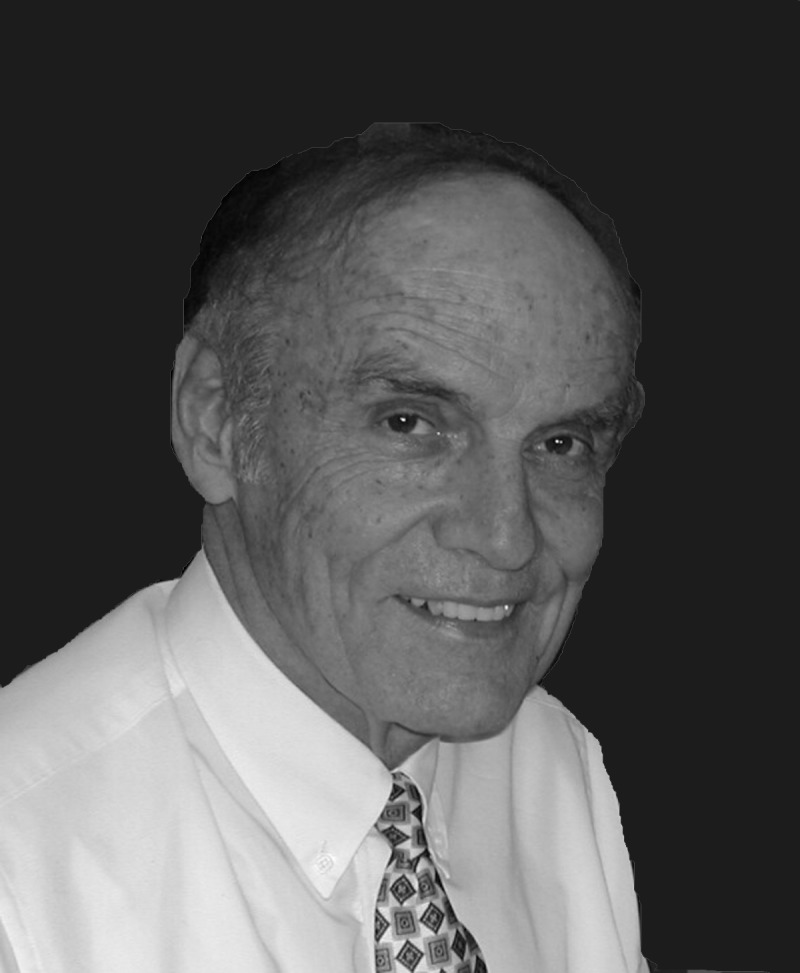
Dr. Halstead is senior advisor and director of research of the pediatric dengue vaccine initiative headquartered at the International Vaccine Institute, Seoul, South Korea. His research interests include the epidemiology, pathogenesis, and vaccinology of flaviviruses.
